# Supplementary material for: Housing structure including the surrounding environment as a risk factor for visceral leishmaniasis transmission in Nepal
Source: PLoS Negl Trop Dis. 2020 Mar 9;14(3):e0008132. doi: 10.1371/journal.pntd.0008132 (PMC7062236; doi:10.1371/journal.pntd.0008132)
Supplement: S1 Checklist — (DOC) [file pntd.0008132.s001.doc]

STROBE Statement—Checklist of items that should be included in reports of ***case-control studies***

|  | Item No | Recommendation |
| --- | --- | --- |
| **Title and abstract** | 1 | (*a*) Indicate the study’s design with a commonly used term in the title or the abstract- The design has been mentioned in the abstract (Abstract, paragraph1) |
|  |  | (*b*) Provide in the abstract an informative and balanced summary of what was done and what was found- It has been provided in the abstract (Abstract, paragraph 1). |
| Introduction | | |
| Background/rationale | 2 | Explain the scientific background and rationale for the investigation being reported- Introduction (paragraphs 1, 2, 3, 4, 5, 6, 7) and introduction (paragraph 8) |
| Objectives | 3 | State specific objectives, including any prespecified hypotheses- Introduction (paragraph 8, 9). |
| Methods | | |
| Study design | 4 | Present key elements of study design early in the paper- Methods, under study design (paragraph 1) |
| Setting | 5 | Describe the setting, locations, and relevant dates, including periods of recruitment, exposure, follow-up, and data collection- Methods study area (paragraph 1), methods data collection procedures (paragraph 1), methods data collection procedures (paragraph 1), no follow up was done. |
| Participants | 6 | (*a*) Give the eligibility criteria, and the sources and methods of case ascertainment and control selection. Give the rationale for the choice of cases and controls- Methods study design (paragraph 1) and data collection procedures (paragraph 1). |
|  |  | (*b*) For matched studies, give matching criteria and the number of controls per case- It is unmatched study. |
| Variables | 7 | Clearly define all outcomes, exposures, predictors, potential confounders, and effect modifiers. Give diagnostic criteria, if applicable- Methods, data collection procedures (paragraph 1). |
| Data sources/ measurement | 8* | For each variable of interest, give sources of data and details of methods of assessment (measurement). Describe comparability of assessment methods if there is more than one group- Methods, data collection procedures (paragraph 1). |
| Bias | 9 | Describe any efforts to address potential sources of bias- No potential bias since we collected data by observation regarding the risk factors of VL. |
| Study size | 10 | Explain how the study size was arrived at- Methods, data collection procedures (paragraph 1). |
| Quantitative variables | 11 | Explain how quantitative variables were handled in the analyses. If applicable, describe which groupings were chosen and why- Methods, data management and analysis (paragraph 1), data collection procedures (paragraph 1). |
| Statistical methods | 12 | (*a*) Describe all statistical methods, including those used to control for confounding- Methods, data management and analysis (paragraph 1) |
|  |  | (*b*) Describe any methods used to examine subgroups and interactions- None |
|  |  | (*c*) Explain how missing data were addressed- Only complete sets of data were used. |
|  |  | (*d*) If applicable, explain how matching of cases and controls was addressed- It is unmatched study. |
|  |  | (*e*) Describe any sensitivity analyses- Not performed. |
| Results | | |
| Participants | 13* | (a) Report numbers of individuals at each stage of study—eg numbers potentially eligible, examined for eligibility, confirmed eligible, included in the study, completing follow-up, and analysed- Tables 1, 2, 3 in results section |
|  |  | (b) Give reasons for non-participation at each stage- No non-participation |
|  |  | (c) Consider use of a flow diagram- Not needed |
| Descriptive data | 14* | (a) Give characteristics of study participants (eg demographic, clinical, social) and information on exposures and potential confounders- Given in tables 1, 2, 3 in results section |
|  |  | (b) Indicate number of participants with missing data for each variable of interest- Not applicable |
| Outcome data | 15* | Report numbers in each exposure category, or summary measures of exposure- Given in results (Tables 1, 2, 3, 4) |
| Main results | 16 | (*a*) Give unadjusted estimates and, if applicable, confounder-adjusted estimates and their precision (e.g, 95% confidence interval). Make clear which confounders were adjusted for and why they were included- Bivariate analysis were given in tables 1, 2, 3) and multivariable analysis (table 4). |
|  |  | (*b*) Report category boundaries when continuous variables were categorized- Given when independent variables are continuous. |
|  |  | (*c*) If relevant, consider translating estimates of relative risk into absolute risk for a meaningful time period- Not applicable |

| Other analyses | 17 | Report other analyses done—eg analyses of subgroups and interactions, and sensitivity analyses- Not used and not required |
| --- | --- | --- |
| Discussion | | |
| Key results | 18 | Summarise key results with reference to study objectives- Discussion (paragraphs 2, 3, 4, 5, 6). |
| Limitations | 19 | Discuss limitations of the study, taking into account sources of potential bias or imprecision. Discuss both direction and magnitude of any potential bias- Discussion (paragraphs 8, 9). |
| Interpretation | 20 | Give a cautious overall interpretation of results considering objectives, limitations, multiplicity of analyses, results from similar studies, and other relevant evidence- Given in discussion (paragraphs 1, 2, 3, 4, 5, 6, 7). |
| Generalisability | 21 | Discuss the generalisability (external validity) of the study results- Given comparing with other evidences (in discussion paragraphs 1, 2, 3, 4, 5, 6, 7) |
| Other information | | |
| Funding | 22 | Give the source of funding and the role of the funders for the present study and, if applicable, for the original study on which the present article is based- Not applicable |

*Give information separately for cases and controls.

**Note:** An Explanation and Elaboration article discusses each checklist item and gives methodological background and published examples of transparent reporting. The STROBE checklist is best used in conjunction with this article (freely available on the Web sites of PLoS Medicine at http://www.plosmedicine.org/, Annals of Internal Medicine at http://www.annals.org/, and Epidemiology at http://www.epidem.com/). Information on the STROBE Initiative is available at http://www.strobe-statement.org.
