# Supplementary material for: Housing structure including the surrounding environment as a risk factor for visceral leishmaniasis transmission in Nepal
Source: PLoS Negl Trop Dis. 2020 Mar 9;14(3):e0008132. doi: 10.1371/journal.pntd.0008132 (PMC7062236; doi:10.1371/journal.pntd.0008132)
Supplement: Attachment — Submitted filename: Response to Reviewer comments.docx [file pntd.0008132.s002.docx]

**Response to Reviewers**

Reviewer #1: Methods seem well handled

**Response:** Thank you.

Reviewer #3: This is an interesting paper approaching the housing structure and the nearby environment as factors associated with the risk of having a VL case in the house during the past five years. Cases (houses with VL cases) were obtained from the surveillance registry of VL cases and controls (houses without VL cases) were randomly selected in the neighborhood of the cases. Some information on the background of the study site and some methodological options used for exploring the study hypothesis were partially or no described as it follows:
1.  The estimated underreporting of VL cases, considering that the registry of cases was made retrospectively;

**Response:** VL notification is compulsory in Nepal. We have shown in another study the long delays of VL cases reporting to the public health services and reporting delays from the district office to the national surveillance system. However, it is generally assumed that at some stage almost all patients get to the health services for diagnosis. Subclinical cases are of course not registered.

2. How could the authors be sure that controls were free of VL cases during the five years period? And what was the rational for choosing a five years period?

**Response:** We asked in control houses for VL/PKDL episodes during the preceding 5 years. In most places they know quite well the symptoms and remember the stay in hospital for treatment. 5 years because this is the period people have a good memory for severe health events. As VL/PKDL are now getting rare, we had to go back for some years.

3. A relatively high proportion of the exposures under exploration could be affected by collinearity issues. There is no mention to such a detail in the methods section, please clarify.

**Response:** We did not find in our statistical analysis significant collinearity among variables of interest.

All three points are now mentioned in the discussion. Many thanks for the advice.
--------------------

Reviewer #1: Interpretation is poor and analysis requires attention.

**Response:** Now, interpretation of the results has been revised in the results section.

Reviewer #3: Results are clear and the exposures were divided into different categories, but there was lacking a rational framework organizing the approach. It seems to me that some exposures could be more proximal to a successful VL transmission case than others. Did the authors think the possibility of organizing an a priori explanatory model to be submitted to the hypothesis testing?

**Response:** We have added in the methods section the rationale /hypothesis for choosing the possible risk factors.

As we know, most of the VL infection cases remained asymptomatic. Then, houses without symptomatic VL cases could have asymptomatic infections.  If it were the case, the whole methodological approach could be questioned. Please consider adding a paragraph on this issue in the discussion section.

**Response:** Many thanks for the suggestions. We have now added a paragraph on asymptomatic cases in the discussion and we have added in the methods section the rationale /hypothesis for choosing the possible risk factors.
--------------------
Reviewer #3: Most of the explanations offered in the discussion section are reasonable points but in fact the methodological approach does not allow definite conclusions on the role of the factors identified as associated to VL transmission. Then, the conclusions should be less emphatic on the practicalities, although all the proposed preventive measures must be implemented for other reasons beyond the VL risk, the scientific conclusions of the paper should be restricted to the facts with their respective limitations.

**Response:** Thank you for the suggestion. We have revised the discussion and conclusion**.**
--------------------

<b>Editorial and Data Presentation Modifications?</b></br><br/>
Use  this section for editorial suggestions as well as relatively minor modifications of existing data that would enhance clarity. If the only modifications needed are minor and/or editorial, you may wish to recommend “Minor Revision” or “Accept”.

Reviewer #1: Major revision

Reviewer #2: (No Response)

Reviewer #3: Line 86 – P. argentipes. Please put the genus name in full.
Line 95. P. argentipes . Please change to the abbreviated genus name.
Line 100. Please consider rephrasing the sentence: “This study compares the housing conditions of previous VL cases and without VL”.
Line 151: Please define immediate environment.

**Response:** Many thanks. Changed as suggested.
--------------------

Summary and General Comments

Reviewer #1: The manuscript deals with an important issue of South Asia that affects huge number of people every year. The authors examined how environmental factors particularly housing structure implicate spread or incidence of VL in Nepal with a case study. Given that climate change and anthropogenic activities could instigate VL vectors into new locations, the study could be useful to develop targeted interventions to save life from VL. So many studies have done in South Asia to map and analyse VL occurrences and there is less room to add new knowledge. Also, there was an elimination program of VL which ran for a decade or more, reading this work gives me an impression that the elimination program possibly did not help much, at least in Nepal. Hence this work could be useful given that the following issues are addressed adequately.
[1] Existing works and review of the VL in South Asia is poorly handled which shows that the motivation of this work is poor.

**Response:** The authors of this paper are working for the last 10 years on the VL elimination initiative on the Indian sub-continent. The revised text has now made reference to the elimination initiative and underlined the relevance for the maintenance phase of the VL elimination.

Therefore there are a few quality works that could help restructuring of the introduction and discussion parts. I am suggesting a few below but more could be obtained from the net via search engine such as Google Scholar.
<https://tropmedhealth.biomedcentral.com/articles/10.1186/s41182-017-0054-9>
<https://www.sciencedirect.com/science/article/pii/S1473309910703200>
<https://www.bmj.com/content/364/bmj.k5224.abstract>
<https://tropmedhealth.biomedcentral.com/articles/10.1186/s41182-017-0069-2>
<https://journals.plos.org/plosntds/article?rev=2&id=10.1371/journal.pntd.0007724>
<https://onlinelibrary.wiley.com/doi/abs/10.1111/tbed.13416>
<https://parasitesandvectors.biomedcentral.com/articles/10.1186/s13071-019-3778-z>

**Response:** The suggested articles were reviewed and we have added references to these articles.

[2] Line 85-90: You are pointing out about environmental change but this part needs really fleshing out more as to how environmental change is affecting the spread of VL in Nepal in particular and in South Asia in general. This would could be useful though <https://link.springer.com/chapter/10.1007/978-3-319-47101-3_19>

**Response:** Now the link to climate and environmental change has been made with reference to the Bangladesh paper.

[3] Line 96–99: good statements but requires more evidence, and above works could provide more evidence.

**Response:** Thanks you for the suggestion. We have added the references.

[4] What is the point of highlighting Table 1? Should be in regular style

**Response:** Thank you. It has been formatted as suggested.

[5] Discussion section: you should relate findings of yours with existing works, what similarities and dissimilarities and why? The results of elimination program also need be highlighted here to show why it worked and why not?

**Response:** Many thanks for the comment. Now references to our and other previous papers in the discussion included. However, we did not expand more on this in this specific paper.

**…………………………………………………………………………….**

Reviewer #2: The present work evaluate variables associated to residences and the environment and relate them to the occurrence of visceral leishmaniasis cases in Nepal.
Despite its importance to local visceral leishmaniasis transmission, i chose to reject the work because i consider the paper do not attend the PLOS NTDs criteria for publication.
One important issue in the decision was the lack of innovation of the work, the application of a predictive model based on mathematicals tools that could influence local control programs and consequently the incidence of cases would strengthen the study. The work is conducted well , with adequate analysis but no novelty in the scientific context.
Some variables evaluated were not observed in the control group and the authors do not make clear whether they were used for multivariate analysis, this may also influence the results.

Another inadequacy is associated with the conclusion that it should propose control measures through the application of a predictive model and not just inform those exposed.
These are some examples that led me to the decision to reject the job. Implementing a predictive model or an extension of the analysis so that environmental findings can be correlated with personal data could strengthen the article in the future.

**Response:** Many thanks for sharing your opinion. We believe that mathematical modelling, if based on sound empirical data, is a very useful complementary tool. Our intention was to collect data with a novel focus on architectural elements. We would be happy to share the data with a modeller to see if he/she will be able to construct a predictive model.

Reviewer #3: This is an original paper on a relevant public health issue. Authors approached an important set of exposures that are vulnerable to preventive interventions. The methodological approach is correct but deserves some improvements related to the description of the analytical procedures and the interpretation of the main results should be better contextualized, considering the study limitations. Please see specific comments above.

**Response:** Many thanks for your review which has helped to improve the text**.** The discussion part has now been partially re-written, the methodological issues mentioned and the interpretation put into context.
--------------------
